# *In vitro* inhibitory effects of Friedelin on human liver cytochrome P450 enzymes

**DOI:** 10.1080/13880209.2018.1491999

**Published:** 2018-08-19

**Authors:** Jinlan Wei, Hongying Zhang, Qingling Zhao

**Affiliations:** a Department of Public Health, Yidu Central Hospital of Weifang, Weifang, Shandong, China;; b Department of Obstetrics, Yidu Central Hospital of Weifang, Weifang, Shandong, China

**Keywords:** CYP3A4, CYP2E1, drug–drug interactions

## Abstract

**Context:** Friedelin is a triterpenoid with several biological activities. However, the affects of Friedelin on the activity of human liver cytochrome P450 (CYP) enzymes remains unclear.

**Objective:** This study investigates the inhibitory effects of Friedelin on the major human liver CYP isoforms (CYP3A4, 1A2, 2A6, 2E1, 2D6, 2C9, 2C19 and 2C8).

**Materials and methods:** First, the inhibitory effects of Friedelin (100 μM) on the eight human liver CYP isoforms were investigated *in vitro* using human liver microsomes (HLMs), and then enzyme inhibition, kinetic studies, and time-dependent inhibition studies were conducted to investigate the IC_50_, *K*_i_ and *K*_inact_/*K*_I_ values of Friedelin.

**Results:** The results indicate that Friedelin inhibited the activity of CYP3A4 and 2E1, with the IC_50_ values of 10.79 and 22.54 μM, respectively, but other CYP isoforms were not affected. Enzyme kinetic studies showed that Friedelin is not only a noncompetitive inhibitor of CYP3A4, but also a competitive inhibitor of CYP2E1, with *K*_i_ values of 6.16 and 18.02 μM, respectively. In addition, Friedelin is a time-dependent inhibitor of CYP3A4 with *K*_inact_/*K*_i_ value of 4.84 nM/min.

**Discussion and conclusion:** The *in vitro* studies of Friedelin with CYP isoforms suggested that Friedelin has the potential to cause pharmacokinetic drug interactions with other co-administered drugs metabolized by CYP3A4 and 2E1. Further clinical studies are needed to evaluate the significance of this interaction.

## Introduction

Friedelin is a triterpenoid isolated from the leaves of *Maytenus ilicifolia* (Mart.) ex. Reissek (Celastraceae) and has several biological activities such as antioxidant, *in vitro*-cytotoxic, antiobesity, anti-inflammatory and antiulcer (Shimizu and Tomoo 1994; Paul et al. 2013; Susanti et al. 2013; Utami et al. 2013). A previous study has reported that Friedelin could suppress ATPase activity of P-glycoprotein (P-gp), which could improve the bioavailability of P-gp substrate when they are co-administered (Lee et al. 2017).

Cytochrome P450 (CYP) enzymes are important phase I enzymes in the biotransformation of xenobiotics, and most of the CYP enzymes can be inhibited or induced by a variety of drugs and chemicals that can give rise to toxicity or treatment failure (Wrighton and Stevens 1992; Li 2001; Yan and Caldwell 2001; Peng et al. 2015). In clinical practice, many patients undergo multiple-drug therapy. Multiple-drug therapy possess several advantages, and however, many herb–drug interactions resulting from concurrent use of herbal drugs with prescription and over-the-counter drugs may cause adverse reactions such as toxicity and treatment failure (Zhang et al. 2007; Grimm et al. 2009; Jeong et al. 2013; Lee et al. 2013; Qi et al. 2013; Meng and Liu 2014). Therefore, the effects of Friedelin on the activity of CYP enzymes should also be investigated. To the best of our knowledge, no studies have been performed to investigate the effects of Friedelin on the major CYP enzymes, particularly the inhibitory effects, which will increase the risk of therapeutic applications of Friedeli and its medical preparations.

This study investigates the effects of Friedelin on eight major CYP isoforms in human liver microsomes (HLMs). *In vitro*, testosterone (CYP3A4), phenacetin (CYP1A2), coumarin (CYP2A6), chlorzoxazone (CYP2E1), dextromethorphan (CYP2D6), diclofenac (CYP2C9), *S*-mephenytoin (CYP2C19) and paclitaxel (CYP2C8) were used as probe substrates to determine the effects of Friedelin on eight CYP enzymes. Also, enzyme kinetic studies were conducted to determine the inhibition mode of Friedelin on CYP enzymes.

## Materials and methods

### Chemicals

Friedelin (≥98%) and testosterone (≥98%) were obtained from the National Institute for the Control of Pharmaceutical and Biological Products (Beijing, China). d-Glucose-6-phosphate, glucose-6-phosphate dehydrogenase, corticosterone (≥98%), NADP^+^, phenacetin (≥98%), acetaminophen (≥98%), 4-hydroxymephenytoin (≥98%), 7-hydroxycoumarin (≥98%), 4′-hydroxydiclofenac (≥98%), sulfaphenazole (≥98%), quinidine (≥98%), tranylcypromine (≥98%), chlorzoxazone (≥98%), 6-hydroxychlorzoxazone (≥98%), paclitaxel (≥98%), 6β-hydroxytestosterone (≥98%), clomethiazole (≥98%), and furafylline (≥98%) were obtained from Sigma Chemical Co. Montelukast (≥98%) was obtained from Beijing Aleznova Pharmaceutical (Beijing, China). Coumarin (≥98%), diclofenac (≥98%), dextromethorphan (≥98%), and ketoconazole (≥98%) were purchased from ICN Biomedicals. Pooled HLMs were purchased from BD Biosciences Discovery Labware. All other reagents and solvents were of analytical reagent grade.

### Assay with human liver microsomes

As shown in Table 1, to investigate the inhibitory effects of Friedelin on different CYP isoforms in HLM, the following probe reactions were used, according to previously described method (Zhang et al. 2007; Qi et al. 2013): testosterone 6β-hydroxylation for CYP3A4, phenacetin *O*-demethylation for CYP1A2, coumarin 7-hydroxylation for CYP2A6, chlorzoxazone 6-hydroxylation for CYP2E1, dextromethorphan *O*-demethylation for CYP2D6, diclofenac 4′-hydroxylation for CYP2C9, *S*-mephenytoin 4-hydroxylation for CYP2C19 and paclitaxel 6α-hydroxylation for CYP2C8. All incubations were performed in triplicate, and the mean values were utilized. The typical incubation systems contained 100 mM potassium phosphate buffer (pH 7.4), NADPH generating system (1 mM NADP^+^, 10 mM glucose-6-phosphate, 1 U/mL of glucose-6-phosphate dehydrogenase, and 4 mM MgCl_2_), the appropriate concentration of HLMs, a corresponding probe substrate and Friedelin (or positive inhibitor for different probe reactions) in a final volume of 200 μL.

**Table 1.** Isoforms tested, marker reactions, incubation conditions, and *K*_m_ used in the inhibition study.

CYPs	Marker reactions	Substrate concentration (μM)	Protein concentration (mg/mL)	Incubation time (min)	Estimated *K*_m_ (μM)
1A2	Phenacetin *O*-deethylation	40	0.2	30	48
3A4	Testosterone 6β-hydroxylation	50	0.5	10	53
2A6	Coumarin 7-hydroxylation	1.0	0.1	10	1.5
2E1	Chlorzoxazone 6-hydroxylation	120	0.4	30	126
2D6	Dextromethorphan *O*-demethylation	25	0.25	20	4.8
2C9	Diclofenac 4'-hydroxylation	10	0.3	10	13
2C19	*S*-Mephenytoin 4-hydroxylation	100	0.2	40	105
2C8	Paclitaxel 6α-hydroxylation	10	0.5	30	16

The concentration of Friedelin was 100 μM, and the positive inhibitor concentrations were as follows: 1 μM ketoconazole for CYP3A4, 10 μM furafylline for CYP1A2, 10 μM tranylcypromine for CYP2A6, 50 μM clomethiazole for CYP2E1, 10 μM quinidine for CYP2D6, 10 μM sulfaphenazole for CYP2C9, 50 μM tranylcypromine for CYP2C19, 5 μM montelukast for CYP2C8. Probe substrates, positive inhibitors (except for dextromethorphan and quinidine which were dissolved in water) and Friedelin were dissolved in methanol, with a final concentration of 1% (v/v) and 1% neat methanol was added to the incubations without inhibitor. The final microsomal protein concentration and incubation times for the different probe reactions are shown in Table 1. There was a 3 min preincubation period (at 37 °C) before the reaction was initiated by adding an NADPH-generating system. The reaction was terminated by adding a 100 μL acetonitrile (10% trichloroacetic acid for CYP2A6) internal standard mix, and the solution was placed on ice. The mixture was centrifuged at 12,000 rpm for 10 min, and an aliquot (50 μL) of the supernatant was transferred for HPLC analysis. The instrument used in this study were Agilent 1260 series instrument with DAD and FLD detector, and the quantitative assay for the corresponding metabolites was performed as previously reported (Lang et al. 2016; Zhang et al. 2017).

### Enzyme inhibition and kinetic studies of Friedelin

Friedelin (100 μM) was used to initially screen for its direct inhibitory effects toward different human CYP isoforms. For the CYP isoforms whose activities were strongly inhibited, secondary studies were performed to obtain the half inhibition concentration (IC_50_). *K*_i_ values were obtained by incubating various concentrations of different probe substrates (20–100 μM testosterone, 25–200 μM chlorzoxazone) in the presence of 0–50 μM Friedelin.

### Time-dependent inhibition study of Friedelin

To determine whether Friedelin could inhibit the activity of CYP3A4 and 2E1 in a time-dependent manner, Friedelin (20 μM) was preincubated with HLMs (1 mg/mL) in the presence of an NADPH-generating system for 30 min at 37 °C. After incubation, an aliquot (20 μL) was transferred to another incubation tube (final volume 200 μL) containing an NADPH-generating system and probe substrates whose final concentrations were approximate to *K*_m_. Then, further incubations were performed to measure the residual activity. After being incubated for different time (0–30 min), the reactions were terminated by adding a 100 μL acetonitrile internal standard mix and then placed on ice; the corresponding metabolites were determined by HPLC.

To determine the *K*_i_ and *k*_inact_ values for the inactivation of CYP3A4, the incubations were conducted using higher probe substrate concentrations (approximately 4-fold *K*_m_ values) and various concentrations of Friedelin (0–50 μM) after different preincubation times (0–30 min), with a two-step incubation scheme, as described above.

### Statistical analysis

The enzyme kinetic parameters for the probe reaction were estimated from the best fit line using least-squares linear regression of the inverse substrate concentration versus the inverse velocity (Lineweaver–Burk plots), and the mean values were used to calculate *V*_max_ and *K*_m_. Inhibition data from the experiments that were conducted using multiple compound concentrations were represented by Dixon plots, and inhibition constant (*K*_i_) values were calculated using nonlinear regression according to the following equation:

$$v=\left(V_{\text{max}}S\right)/\left(K_{m}\left(1+I/K_{i}\right)+S\right),$$

where *I* is the concentration of the compound, *K*_i_ is the inhibition constant, *S* is the concentration of the substrate, and *K*_m_ is the substrate concentration at half the maximum velocity (*V*_max_) of the reaction. The mechanism of the inhibition was inspected using the Lineweaver–Burk plots and the enzyme inhibition models. The data comparison was performed using Student’s *t* test and performed using IBM SPSS statistics 20 (SPSS Inc.).

## Results

**Figure 1.**
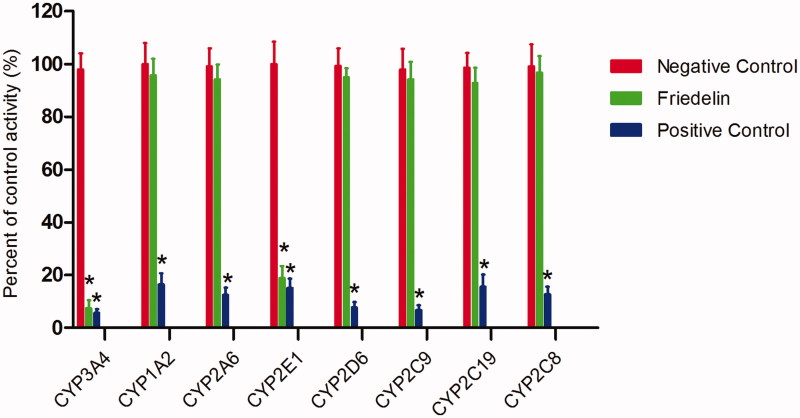
Effects of Friedelin (100 μM) on the activity of CYP450 enzymes in pooled HLMs. All data represent mean ± SD of the triplicate incubations. *p < 0.05, significantly different from the negative control. Negative control: incubation systems without Friedelin; Friedelin: incubation systems with Friedelin; positive control: incubation systems with their corresponding positive inhibitors.

To investigate whether Friedelin affects the catalytic activity of CYP enzymes, the probe reaction assays were conducted with varying concentrations of Friedelin. Specific inhibitors of CYP3A4, 1A2, 2A6, 2E1, 2D6, 2C9, 2C19 and 2C8 were used as positive controls. As shown in Figure 1, Friedelin could not inhibit the activities of CYP1A2, 2A6, 2D6, 2C9, 2C19 and 2C8 at a concentration of 100 μM. In contrast, the activities of CYP3A4 and 2E1 were inhibited to 7.5 and 18.9% of their control activities, respectively.

The enzyme-inhibition study showed that inhibition of CYP3A4 and 2E1 by Friedelin was concentration-dependent, with IC_50_ values of 10.79 and 22.54 μM, respectively.

**Figure 2.**
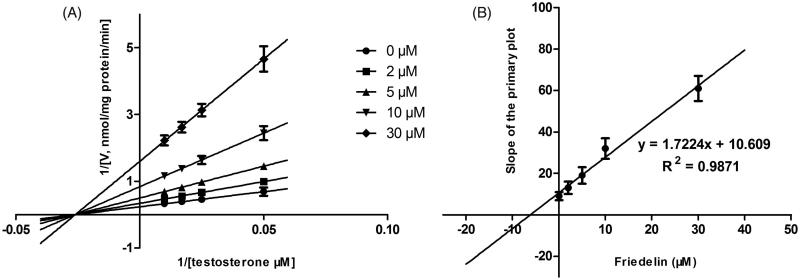
Lineweaver–Burk plots (A) and the secondary plot for *K*_i_ (B) of effects of Friedelin on CYP3A4 catalyzed reactions (testosterone 6β-hydroxylation) in pooled HLM. Data are obtained from a 30 min incubation with testosterone (20–100 μM) in the absence or presence of Friedelin (0–30 μM). All data represent mean ± SD of the triplicate incubations.

**Figure 3.**
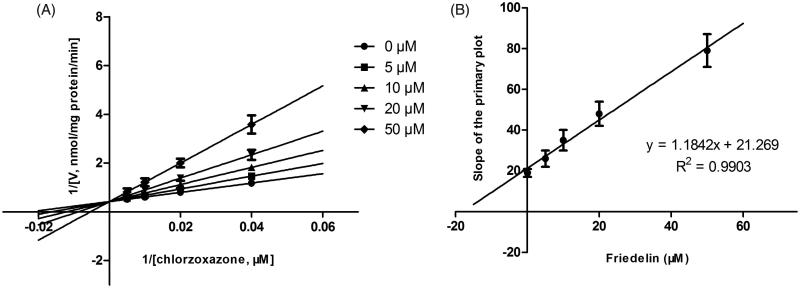
Lineweaver–Burk plots (A) and the secondary plot for *K*_i_ (B) of effects of Friedelin on CYP2E1 catalyzed reactions (chlorzoxazone 6-hydroxylation) in pooled HLM. Data are obtained from a 30 min incubation with chlorzoxazone (25–200 μM) in the absence or presence of Friedelin (0–50 μM). All data represent mean ± SD of the triplicate incubations.

Lineweaver–Burk plots of inhibitory kinetic data suggested that the inhibition of CYP3A4 by Friedelin was best fit in a noncompetitive manner (Figure 2(A)), whereas the inhibition of CYP2E1 (Figure 3(A)) by Friedelin was best fit in a competitive manner. The *K*_i_ values of Friedelin on CYP3A4 (Figure 2(B)) and 2E1 (Figure 3(B)) were obtained from the secondary Lineweaver–Burk plot for *K*_i_, with values of 6.16 and 18.02 μM, respectively.

**Figure 4.**
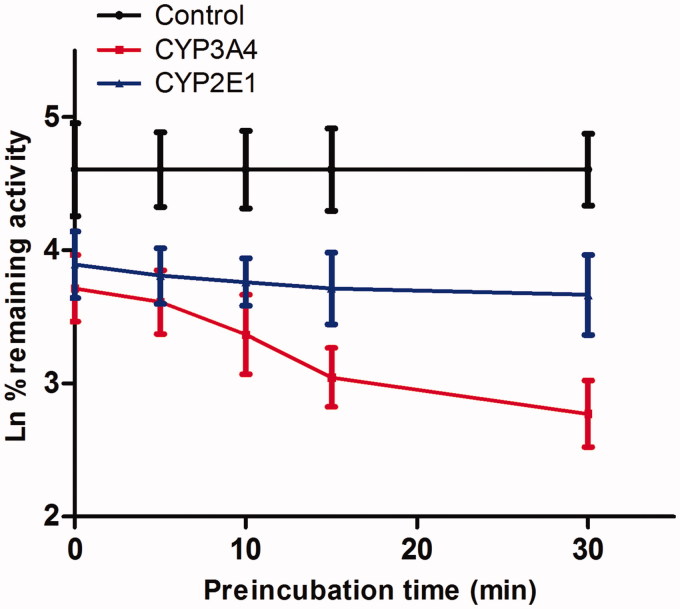
Time-dependent inhibition investigations of CYP3A4 catalyzed testosterone 6β-hydroxylation and CYP2E1 catalyzed chlorzoxazone 6-hydroxylation reactions by Friedelin (20 μM). All data represent mean ± SD of the triplicate incubations.

**Figure 5.**
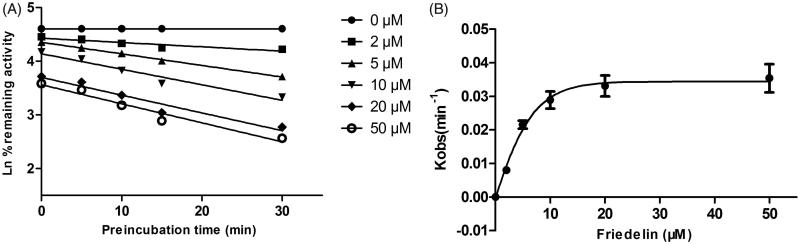
Time and concentration-inactivation of microsomal CYP3A4 catalyzed testosterone 6β-hydroxylation by Friedelin in the presence of NADPH. The initial rate constant of inactivation of CYP3A4 by each concentration (*K*_obs_) was determined through linear regression analysis of the natural logarithm of the percentage of remaining activity versus preincubation time (A). The *K*_i_ and *K*_inact_ values were determined through nonlinear analysis of the *K*_obs_ versus the Friedelin concentration (B).

As shown in Figure 4, after preincubation of Friedelin with HLM for 30 min, the activity of CYP3A4 decreased with the incubation time. However, the activity of CYP2E1 was not affected. To characterize the time-dependent inhibition of CYP3A4 by Friedelin, inactivation parameters of *K*_i_ and *K*_inact_ values were calculated using nonlinear regression analysis in HLM. As calculated from the inactivation plot of Figure 5, the *K*_inact_/*K*_i_ value for CYP3A4 was 4.84 nM/min. The *K*_inact_ values imply that approximately 5.9% of CYP3A4 is inactivated each minute when a saturating concentration of Friedelin is incubated with HLM (Qi et al. 2013).

## Discussion

The most common causes of herb–drug interactions are modification of the enzyme activity of cytochrome P450 enzymes, specifically through inhibitory effects. Inhibition of CYP enzymes *in vivo* may result in unexpected elevations in the plasma concentrations of concomitant drugs, leading to adverse effects (Hu et al. 2015; Liu et al. 2015). Investigating the inhibition mechanisms of Friedelin on CYP isoforms will improve its therapeutic applications and decrease the potential risk of unfavorable herb–drug interactions.

As Friedelin possesses numerous pharmacological activities, has a wide range of applications in the clinic, it is essential to investigate the inhibitory effects of Friedelin on the major CYP enzymes. To the best of our knowledge, this study is the first to investigate the effects of Friedelin on the metabolism of probe substrates of several CYP isoforms, including CYP3A4, 1A2, 2A6, 2E1, 2D6, 2C9, 2C19 and 2C8.

The CYP3A subfamily is one of the dominant CYP enzymes in the liver and extra-hepatic tissues, such as the intestines, and it plays an important role in the oxidation of xenobiotics and contributes to the biotransformation of approximately 60% of currently used therapeutic drugs (Pandit et al. 2011). Human CYP3A4 is one of the most abundant drug-metabolizing CYP isoforms in human liver microsomes, accounting for approximately 40% of the total CYP enzymes (Zhou 2008). In fact, characterization of the CYP3A4 isoform responsible for the metabolism of drugs and herbal constituents is important for identifying potential drug–drug or herb–drug interactions in humans. The present study showed that Friedelin had inhibitory effects *in vitro* on CYP3A4 isoform, with *K*_i_ and IC_50_ values of 6.16 and 10.79 μM, respectively. The results suggested that Friedelin was also a weak CYP3A4 inhibitor, and the potential of herb–drug interaction with CYP3A4 would also be low. However, the results also indicated that Friedelin is a time-dependent inhibitor for CYP3A4 with *K*_inact_/*K*_i_ value of 4.84 nM/min, which revealed that Friedelin would inhibit the activity of CYP3A4 with the increase of preincubation time. Therefore, to avoid adverse drug interactions, Friedelin should not be used with other drugs metabolized by CYP3A4.

CYP2E1 also plays an important role in the metabolism of many drugs (Nowack 2008). Our study showed that Friedelin competitively inhibited human liver microsomal CYP2E1 activity. Therefore, Friedelin should also be used carefully with drugs metabolized by CYP2E1 to avoid possible drug interactions.

As we know, *in vitro* data are essential for understanding a potential enzyme inhibition and DDI *in vivo*. However, an observed *in vitro* inhibition of a CYP enzyme does not mean that the drug will cause clinically relevant interactions. Many other factors might influence drug interactions mediated by CYP inhibition, including the contribution of the hepatic clearance to the total clearance of the affected drug, the fraction of the hepatic clearance which is subject to metabolic inhibition, and the ratio of the inhibition constant (*K*_i_) over the *in vivo* concentration of the inhibitor (Ito et al. 1998; Ericsson et al. 2014). Therefore, further *in vivo* system studies are needed to identify the interactions of Friedelin with CYP isoform in humans.

The results of this study indicate that Friedelin may influence the *in vitro* metabolism of drugs that are substrates of CYP3A4 and 2E1, and therefore, herb–drug interaction might occur when Friedelin is co-administered with the substrates of the CYP3A4 and 2E1. However, there is little data available for the *in vivo* pharmacokinetic data of Friedelin, and therefore, the herb-drug interaction between Friedelin and other drugs is still difficult to predict. Due to the pharmacological activities of Friedelin, further pharmacokinetic studies should be conducted.

In conclusion, the effects of Friedelin on the activity of the major CYP enzymes were investigated *in vitro*. The results showed that Friedelin could inhibit the activity of CYP3A4 and 2E1, while the activity of other CYP enzymes was not affected. Therefore, to avoid adverse herb-drug interactions, caution should be exercised when Friedelin is co-administered with other drugs metabolized by CYP3A4 and 2E1.

## References

[CIT0001] Ericsson T, Sundell J, Torkelsson A, Hoffmann KJ, Ashton M 2014 Effects of artemisinin antimalarials on cytochrome P450 enzymes *in vitro* using recombinant enzymes and human liver microsomes: potential implications for combination therapies. Xenobiotica. 44:615–626. 24400699 10.3109/00498254.2013.878815

[CIT0002] Grimm SW, Einolf HJ, Hall SD, He K, Lim HK, Ling KH, Lu C, Nomeir AA, Seibert E, Skordos KW, et al. 2009 The conduct of *in vitro* studies to address time-dependent inhibition of drug-metabolizing enzymes: a perspective of the pharmaceutical research and manufacturers of America. Drug Metab Dispos. 37:1355–1370. 19359406 10.1124/dmd.109.026716

[CIT0003] Hu X, Huang W, Yang Y 2015 Cytochrome P450 isoenzymes in rat and human liver microsomes associate with the metabolism of total coumarins in *Fructus Cnidii*. Eur J Drug Metab Pharmacokinet. 40:373–377. 24993184 10.1007/s13318-014-0219-4

[CIT0004] Ito K, Iwatsubo T, Kanamitsu S, Nakajima Y, Sugiyama Y 1998 Quantitative prediction of *in vivo* drug clearance and drug interactions from *in vitro* data on metabolism, together with binding and transport. Annu Rev Pharmacol Toxicol. 38:461–499. 9597163 10.1146/annurev.pharmtox.38.1.461

[CIT0005] Jeong HU, Kong TY, Kwon SS, Hong SW, Yeon SH, Choi JH, Lee JY, Cho YY, Lee HS 2013 Effect of honokiol on cytochrome P450 and UDP-glucuronosyltransferase enzyme activities in human liver microsomes. Molecules (Basel, Switzerland). 18:10681–10693. 10.3390/molecules180910681 PMC6269737 24005963

[CIT0006] Lang J, Li W, Zhao J, Wang K, Chen D 2016 Inhibitory effects of curculigoside on human liver cytochrome P450 enzymes. Xenobiotica. 47:849–855. 10.1080/00498254.2016.1257171 27819189

[CIT0007] Lee JA, Ha SK, Kim YC, Choi I 2017 Effects of Friedelin on the intestinal permeability and bioavailability of apigenin. Pharmacol Rep. 69:1044–1048. 28939344 10.1016/j.pharep.2017.04.012

[CIT0008] Lee SY, Lee JY, Kang W, Kwon KI, Park SK, Oh SJ, Ma JY, Kim SK 2013 Cytochrome P450-mediated herb–drug interaction potential of Galgeun-tang. Food Chem Toxicol. 51:343–349. 23104244 10.1016/j.fct.2012.10.012

[CIT0009] Li AP 2001 Screening for human ADME/Tox drug properties in drug discovery. Drug Discov Today. 6:357–366. 11267922 10.1016/s1359-6446(01)01712-3

[CIT0010] Liu T, Qian G, Wang W, Zhang Y 2015 Molecular docking to understand the metabolic behavior of GNF-351 by CYP3A4 and its potential drug–drug interaction with ketoconazole. Eur J Drug Metab Pharmacokinet. 40:235–238. 24756863 10.1007/s13318-014-0201-1

[CIT0011] Meng Q, Liu K 2014 Pharmacokinetic interactions between herbal medicines and prescribed drugs: focus on drug metabolic enzymes and transporters. Curr Drug Metab. 15:791–807. 25705905 10.2174/1389200216666150223152348

[CIT0012] Nowack R 2008 Review article: cytochrome P450 enzyme, and transport protein mediated herb–drug interactions in renal transplant patients: grapefruit juice, St John's Wort - and beyond!. Nephrology (Carlton). 13:337–347. 18363644 10.1111/j.1440-1797.2008.00940.x

[CIT0013] Pandit S, Mukherjee PK, Ponnusankar S, Venkatesh M, Srikanth N 2011 Metabolism mediated interaction of α-asarone and *Acorus calamus* with CYP3A4 and CYP2D6. Fitoterapia. 82:369–374. 21062640 10.1016/j.fitote.2010.11.009

[CIT0014] Paul J, Gnanam R, Jayadeepa RM, Arul L 2013 Anticancer activity on Graviola, an exciting medicinal plant extract vs. various cancer cell lines and a detailed computational study on its potent anti-cancerous leads. Curr Top Med Chem. 13:1666–1673. 23889049 10.2174/15680266113139990117

[CIT0015] Peng Y, Wu H, Zhang X, Zhang F, Qi H, Zhong Y, Wang Y, Sang H, Wang G, Sun J 2015 A comprehensive assay for nine major cytochrome P450 enzymes activities with 16 probe reactions on human liver microsomes by a single LC/MS/MS run to support reliable *in vitro* inhibitory drug–drug interaction evaluation. Xenobiotica 45:961–977. 26007223 10.3109/00498254.2015.1036954

[CIT0016] Qi XY, Liang SC, Ge GB, Liu Y, Dong PP, Zhang JW, Wang AX, Hou J, Zhu LL, Yang L, et al. 2013 Inhibitory effects of sanguinarine on human liver cytochrome P450 enzymes. Food Chem Toxicol. 56:392–397. 23500771 10.1016/j.fct.2013.02.054

[CIT0017] Shimizu M, Tomoo T 1994 Anti-inflammatory constituents of topically applied crude drugs. V. Constituents and anti-inflammatory effect of Aoki, *Aucuba japonica* Thunb. Biol Pharm Bull. 17:665–667. 7920429 10.1248/bpb.17.665

[CIT0018] Susanti D, Amiroudine MZ, Rezali MF, Taher M 2013 Friedelin and lanosterol from *Garcinia prainiana* stimulated glucose uptake and adipocytes differentiation in 3T3–L1 adipocytes. Nat Prod Res. 27:417–424. 22988818 10.1080/14786419.2012.725399

[CIT0019] Utami R, Khalid N, Sukari MA, Rahmani M, Abdul AB. Dachriyanus 2013 Phenolic contents, antioxidant and cytotoxic activities of *Elaeocarpus floribundus* Blume. Pak J Pharm Sci. 26:245–250. 23455191

[CIT0020] Wrighton SA, Stevens JC 1992 The human hepatic cytochromes P450 involved in drug metabolism. Crit Rev Toxicol. 22:1–21. 1616599 10.3109/10408449209145319

[CIT0021] Yan Z, Caldwell GW 2001 Metabolism profiling, and cytochrome P450 inhibition and induction in drug discovery. Curr Top Med Chem. 1:403–425. 11899105 10.2174/1568026013395001

[CIT0022] Zhang H, Ya G, Rui H 2017 Inhibitory effects of triptolide on human liver cytochrome P450 enzymes and P-glycoprotein. Eur J Drug Metab Pharmacokinet. 42:89–98. 26874845 10.1007/s13318-016-0323-8

[CIT0023] Zhang JW, Liu Y, Cheng J, Li W, Ma H, Liu HT, Sun J, Wang LM, He YQ, Wang Y, et al. 2007 Inhibition of human liver cytochrome P450 by star fruit juice. J Pharm Pharm Sci. 10:496–503. 18261370 10.18433/j30593

[CIT0024] Zhou SF 2008 Drugs behave as substrates, inhibitors and inducers of human cytochrome P450 3A4. Curr Drug Metab. 9:310–322. 18473749 10.2174/138920008784220664

